# Predictive role of shock index in the early formation of cerebral infarction in patients with TBI and cerebral herniation

**DOI:** 10.3389/fneur.2022.956039

**Published:** 2022-08-25

**Authors:** Xiaofang Hu, Jun Tian, Jinhua Xie, Shaorui Zheng, Liangfeng Wei, Lin Zhao, Shousen Wang

**Affiliations:** Department of Neurosurgery, The 900th Hospital, Fuzhou, China

**Keywords:** traumatic brain injury, brain herniation, post-traumatic cerebral infarction (PTCI), risk factors, shock index (SI)

## Abstract

**Background and purpose:**

Traumatic brain injury (TBI) with brain herniation predisposes to posttraumatic cerebral infarction (PTCI), which in turn seriously affects the prognosis of patients. At present, there is a lack of effective indicators that can accurately predict the occurrence of PTCI. We aimed to find possible risk factors for the development of PTCI by comparing the preoperative and postoperative clinical data of TBI patients with brain herniation.

**Methods:**

The clinical data of 120 patients with craniocerebral trauma and brain herniation were retrospectively analyzed. Among them, 54 patients had cerebral infarction within 3–7 days after injury. The two groups of patients were compared through univariate and multivariate logistic regression analysis, and a classification tree model and a nomogram model were constructed. Finally, receiver operating characteristic curve analysis and decision curve analysis were conducted to analyze the clinical utility of the prediction model.

**Results:**

Logistic regression analysis showed that factors like the Glasgow Coma Scale (GCS) score (*P* = 0.002), subarachnoid hemorrhage (SAH) (*P* = 0.005), aspiration pneumonia (*P* < 0.001), decompressive craniectomy (*P* < 0.05), intracranial pressure (ICP) monitoring (*P* = 0.006), the shock index (SI) (*P* < 0.001), the mean arterial pressure (MAP) (*P* = 0.005), and blood glucose (GLU) (*P* < 0.011) appeared to show a significant statistical correlation with the occurrence of infarction (*P* < 0.05), while age, sex, body temperature (T), D-dimer levels, and coagulation tests were not significantly correlated with PTCI after cerebral herniation. Combined with the above factors, Classification and Regression Tree was established, and the recognition accuracy rate reached 76.67%.

**Conclusions:**

GCS score at admission, no decompressive craniectomy, no ICP monitoring, combined SAH, combined aspiration pneumonia, SI, MAP, and high GLU were risk factors for infarction, of which SI was the primary predictor of PTCI in TBI with an area under the curve of 0.775 (95% CI = 0.689–0.861). Further large-scale studies are needed to confirm these results.

## Introduction

Brain herniation is one of the most serious complications of craniocerebral trauma. Patients with brain herniation often suffer from intracranial lesions and simultaneous or habitual damage to other organs. Posttraumatic cerebral infarction (PTCI) is one of the most common secondary lesions in patients with cerebral herniation (1). The incidence rate in the first 2 weeks after injury is as high as 71.6% (2). The lesions are mostly seen in the formation of brain tissue resulting from brain hernia, mainly due to the increase of intracranial pressure (ICP) caused by brain injury, blood supply disorder caused by insufficient perfusion in parts of the brain (3), and the selective loss of damaged neurons, resulting in neurological dysfunction. Patients with craniocerebral trauma often have multiple injuries such as cerebral contusion, intracranial hematoma, and traumatic subarachnoid hemorrhage (SAH), which are often accompanied by disturbance of consciousness upon admission, and it is very difficult to diagnose PTCI at an early stage (4–6). The prognosis and mortality of patients with PTCI are higher than those of patients without infarction. In the past, it was thought that the incidence of PTCI after traumatic brain injury (TBI) was lower, but recent studies have shown that its incidence lies between 8 and 19.1% (7). In the autopsy of patients who died of severe craniocerebral trauma, 60–90% show pathological features of ischemic brain injury (8).

There are many pathogenic factors for cerebral infarction secondary to brain hernia after craniocerebral injury. In the present study, we retrospectively analyzed 120 patients with traumatic extra severe craniocerebral injury complicated with brain hernia in the Neurosurgery Department of 900th Hospital from January 2018 to January 2020. This study was conducted to explore whether TBI with brain herniation predisposes to PTCI and to construct a classification tree and conduct logistic regression analysis to identify the influencing factors for quantitative analysis, in order to evaluate and predict the risk of cerebral infarction in patients with TBI and to improve prognosis.

## Materials and methods

### Study population

This was an observational, retrospective cohort study. A total of 167 patients with craniocerebral trauma admitted to the Neurosurgery Department of the 900th Hospital between January 2018 and January 2020 were collected. All patients underwent surgery, which was performed by physicians in the trauma treatment group. The inclusion criteria were as follows: (1) patients with posttraumatic brain herniation were admitted to the hospital, and the Glasgow Coma Scale (GCS) score was 3–8; (2) head CT on admission showed midline shift and compression of ventricle structures at admission; and (3) patients were admitted to the hospital within 6 h after injury. Exclusion criteria were as follows: (1) non-cerebral infarct formation in patients with TBI; (2) bilateral mydriasis without spontaneous breathing and no indication for surgery; (3) patients aged <20 years or >70 years; (4) patients who died immediately after admission; (5) Patients with multiple surgeries; (6) a history of atrial fibrillation, cerebral infarction, venous thrombosis, liver disease, and/or blood and other diseases; and (7) a history of taking anticoagulant drugs. On the basis of these criteria, 47 patients were excluded.

### Basic characteristics of the population

**Figure 1:**
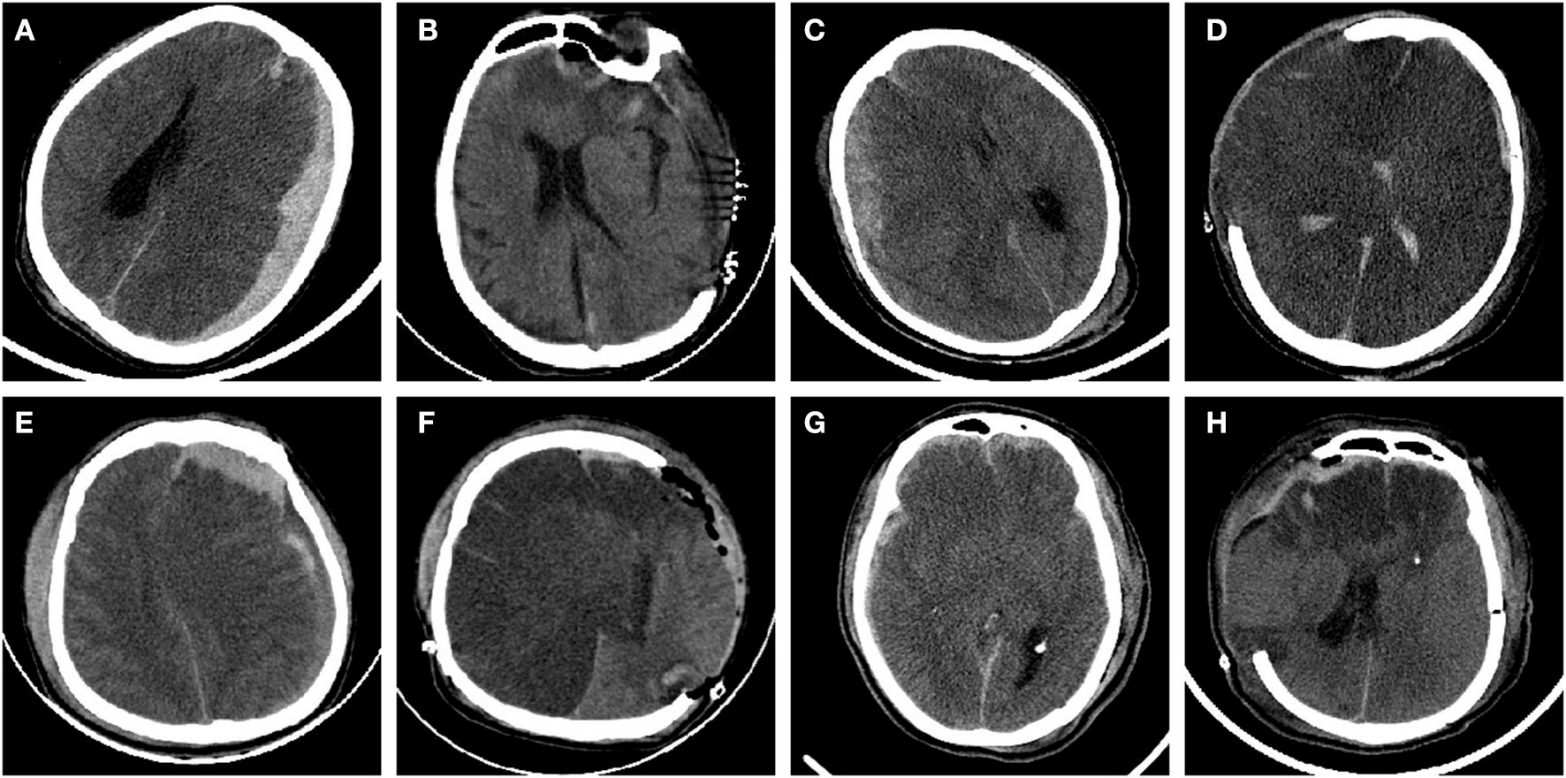
CT imaging of patients with secondary cerebral infarction after TBI cerebral hernia. **(A)** Left frontotemporoparietal hematoma after TBI. **(B)** Hematoma evacuation and decompressive craniectomy showing a left occipital low-density infarction area. **(C)** Right frontotemporal hematoma with SAH. **(D)** Decompressive craniectomy showing cerebral hemispheric infarction. **(E)** Left frontotemporoparietal epidural hematoma with arachnoid hemorrhage. **(F)** Hematoma evacuation and decompressive craniectomy showed extensive infarction of the right cerebral hemisphere. **(G)** Frontal lobe laceration, right frontotemporal hematoma, and arachnoid hemorrhage. **(H)** Postoperative frontal lobe infarction.

All 120 enrolled TBI patients had brain herniation. Among these patients, there were 89 cases of cerebral contusion, 45 cases of diffuse axonal injury, 89 cases of intracranial hematoma, 43 cases of epidural hematoma, 67 cases of subdural hematoma, and 72 cases of SAH. The above types of cerebral injury were present alone or in combination in the included cases. The causes of injury include: traffic accidents, elevated fall injuries, and impact injuries. Dynamic review of head CT showed cerebral infarction in unilateral frontal lobe, temporal lobe, occipital lobe, unilateral cerebral hemisphere or bilateral infarction within 1 week after injury (Figure 1).

### Statistical analysis

SPSS 23.0 software was used for statistical analysis. Categorical data are presented as frequency and percentage, and continuous variables are presented as mean ± standard deviation. Univariate and multivariate logistic regression analysis methods were used to analyze the influencing factors before treatment. In our multivariate analysis, we applied variable criterion *P* = 0.05 and exclusion criterion *P* = 0.10. A Classification and Regression Tree was constructed by applying independent variables, and the significance of the independent variables was analyzed. The effect of model fitting was judged by ROC curves. At the same time, a Alignment Diagram was established and DCA curves were drawn to assess whether the risk of PTCI occurred.

## Results

### Patient characteristics

In total, 120 patients were enrolled, including 93 males (80%) and 37 females (20%). Based on 28-day survival, patients were divided into the survival group and the death group. To analyze whether there was an association between infarction and the risk of death. The results are as follows (Table 1).

**Table 1:** The relationship between cerebral infarction and death.

**Variable**	**The survival** **group**	**The death** **group**	***P*-value**	**OR (95% CI)**
**Cerebral infarction**				
None	43	23	<0.001	4.069 (1.892~8.750)
Yes	17	37		

*From the above, it can be seen that there was a significant difference in the risk of death between the infarction group and the control group. The risk of death was 4.069 times higher in the infarction group*.

### Risk factors of PTCI

With cerebral infarction as the dependent variable, univariate logistic regression analysis with the following independent variables: sex, age, SAH, aspiration pneumonia, decompressive craniectomy (DC), mean arterial pressure (MAP), GCS score, blood glucose (GLU), the shock index (SI), and ICP monitoring. The results showed that SAH, aspiration pneumonia, DC, MAP, GCS, GLU, SI, and ICP were significantly associated with the occurrence of PTCI (Table 2).

**Table 2:** Univariate analysis of PTCI-related variables in 120 patients with TBI and herniation.

**Variable**	**Classification**	**None-cerebral infarction (%)**	**Cerebral infarction (%)**	***P*-value**	**OR (95% CI)**
Gender	Male	51 (53.12)	45 (46.88)	0.411	0.680 (0.271~1.704)
	Female	15 (62.50)	9 (37.50)		
Age		53.42 ± 19.76	58.54 ± 12.23	0.103	1.019 (0.996~1.042)
SAH	None	34 (70.83)	14 (29.17)	**0.005**	3.036 (1.396~6.601)
	Yes	32 (44.44)	40 (55.56)		
Aspiration pneumonia	None	54 (75.00)	18 (25.00)	**<0.001**	9.000 (3.872~20.919)
	Yes	12 (25.00)	36 (75.00)		
DC	None	23 (36.51)	40 (63.49)		
	Yes	43 (75.44)	14 (24.56)	**<0.001**	5.342 (2.420~11.790)
MAP		95.08 ± 19.19	86.99 ± 15.70	**0.017**	0.974 (0.957~0.995)
GCS		6.09 ± 3.64	4.32 ± 1.41	**0.002**	0.765 (0.640~0.914)
GLU		11.74 ± 5.19	14.74 ± 7.15	**0.011**	1.083 (1.018~1.152)
SI		0.72 ± 0.28	1.00 ± 0.29	**<0.001**	27.539 (6.141~123.496)
ICP monitoring	None	38 (46.34%)	44 (53.66%)	**0.006**	0.308 (0.133~0.716)
	Yes	28 (73.68%)	10 (26.32%)		
D-Dimer		22.64 ± 13.82	25.63 ± 13.65	0.235	1.016 (0.990~1.044)
PT		20.67 ± 30.17	16.23 ± 9.51	0.337	0.990 (0.969~1.011)
INR		3.31 ± 15.35	1.45 ± 0.90	0.585	0.970 (0.869~1.082)
APTT		34.00 ± 15.64	33.46 ± 14.10	0.840	0.997 (0.973~1.022)
TT		24.12 ± 15.79	22.78 ± 10.04	0.590	0.992 (0.965~1.021)
Temperature		37.54 ± 1.07	37.26 ± 4.94	0.669	0.976 (0.874~1.090)

*SAH, subarachnoid hemorrhage; DC, decompressive craniectomy; MAP, mean arterial pressure; GCS, Glasgow Coma Scale; GLU, blood glucose; SI, shock index; ICP, intracranial pressure; PT, prothrombin time; APTT, activated partial thromboplastin time; TT, thrombin time*.

*The bold values represent p < 0.05 which is statistically significant*.

### Multivariate regression analysis of PTCI

Factors that were significantly correlated with PTCI were included in our multivariate logistic regression analysis. The results showed that SAH, aspiration pneumonia, DC, and SI were risk factors for TBI. Among them, the risk of TBI with SAH was 3.2-fold higher than that without SAH, and the risk of TBI with aspiration pneumonia was 8.6-fold higher than that without aspiration pneumonia. It was also found that higher SI was associated with a higher risk of PTCI. In addition, monitoring of DC and ICP was a protective factor for PTCI (Table 3).

**Table 3:** Multivariate analysis of the risk of PTCI.

**Variable**	**B**	**Wald**	***P*-value**	**OR value**	**95% CI**
SAH	1.155	4.091	0.043	3.172	1.036~9.711
Aspiration pneumonia	2.154	15.085	0.000	8.617	2.906~25.548
DC	1.833	10.636	0.001	6.255	2.078~18.826
SI	2.466	5.460	0.019	11.770	1.488~93.094
ICP monitoring	−1.250	4.172	0.041	0.287	0.086~0.951

*SAH, subarachnoid hemorrhage; DC, decompressive craniectomy; SI, shock index; ICP, intracranial pressure*.

### Classification and regression tree model and ROC curve analysis

**Figure 2:**
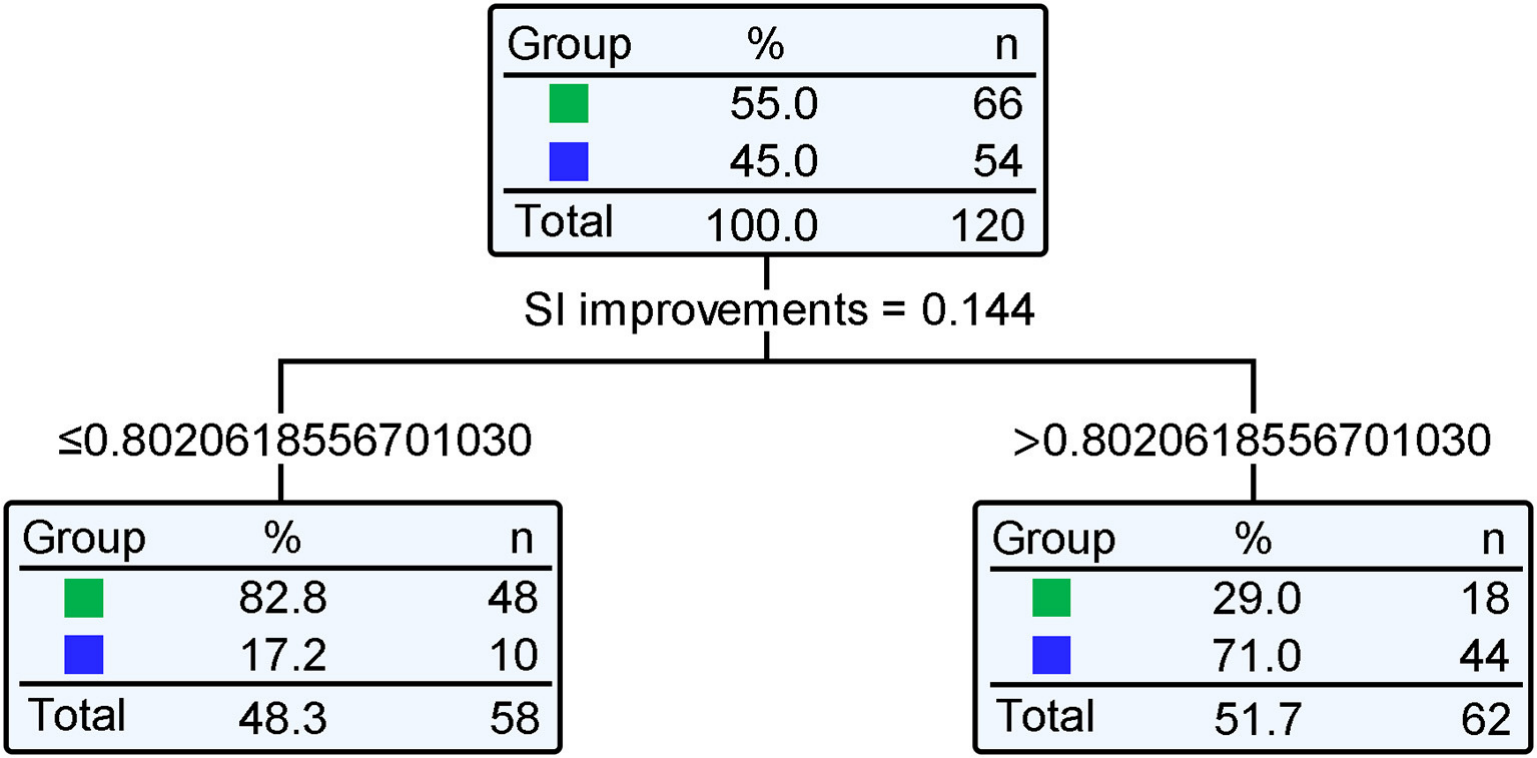
Classification and Regression Tree of the risk of cerebral hernia patients with PTCI. Standardized importance analysis showed that SI was the primary influencing factor for PTCI. Taking SI as the independent variable and cerebral infarction as the dependent variable, the predictive value of cerebral infarction in patients with traumatic brain hernia showed that the optimal SI cut-off point was 0.802.

**Figure 3:**
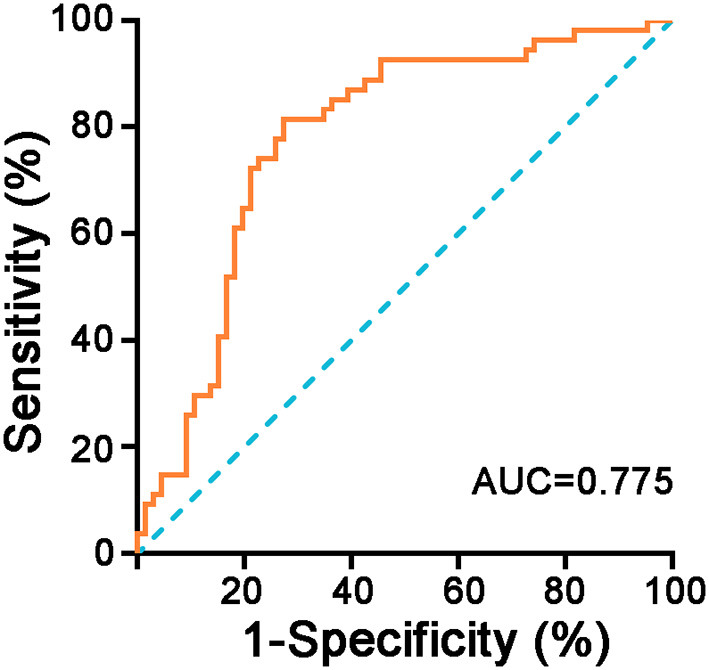
ROC curve of SI for predicting PTCI in patients with TBI brain herniation. The receiver operating characteristic (ROC) curve showed that the area under the curve was 0.775 (95% CI = 0.689–0.861, *P* < 0.001).

A Classification and Regression Tree was constructed with variables such as SAH, aspiration pneumonia, DC, SI, and ICP monitoring. The model's recognition accuracy rate reached 76.67%. Next, risk assessment of the classification and regression tree model was carried out. The proportion of incorrect classifications of the model as determined by the cross-validation method was 23.33%, and the standard error was 0.039, indicating that the model fitting was good. Standardized importance analysis showed that SI was the primary influencing factor for PTCI (Figure 2); compared with SI, the standardized importance of aspiration pneumonia, DC, SAH, and ICP monitoring was 83.2, 52.4, 23.2, and 22.4%, respectively (Table 4). Taking SI as the independent variable and cerebral infarction as the dependent variable, the ROC curve showed that the area under the curve was 0.775 (95% CI = 0.689–0.861) (*P* < 0.001). The cut-off value is 0.802, and the obtained sensitivity, specificity, and Youden index were all high, indicating that the SI classification results had practical significance, and the classification effect was good (Figure 3), with high predictive value.

**Table 4:** Importance of independent variables.

**Independent variable**	**Importance**	**Importance of standardization**
SI	0.144	100.0%
Aspiration pneumonia	0.120	83.2%
DC	0.076	52.4%
Subarachnoid hemorrhage	0.033	23.2%
ICP monitoring	0.032	22.4%

*Compared with SI, the standardized importance of aspiration pneumonia, DC, SAH, and ICP monitoring were 83.2, 52.4, 23.2, and 22.4%, respectively. Standardized importance analysis showed that SI was the primary predictor of PTCI*.

### Establishment of a nomographic model for predicting the risk of PTCI in patients with cerebral herniation

**Figure 4:**
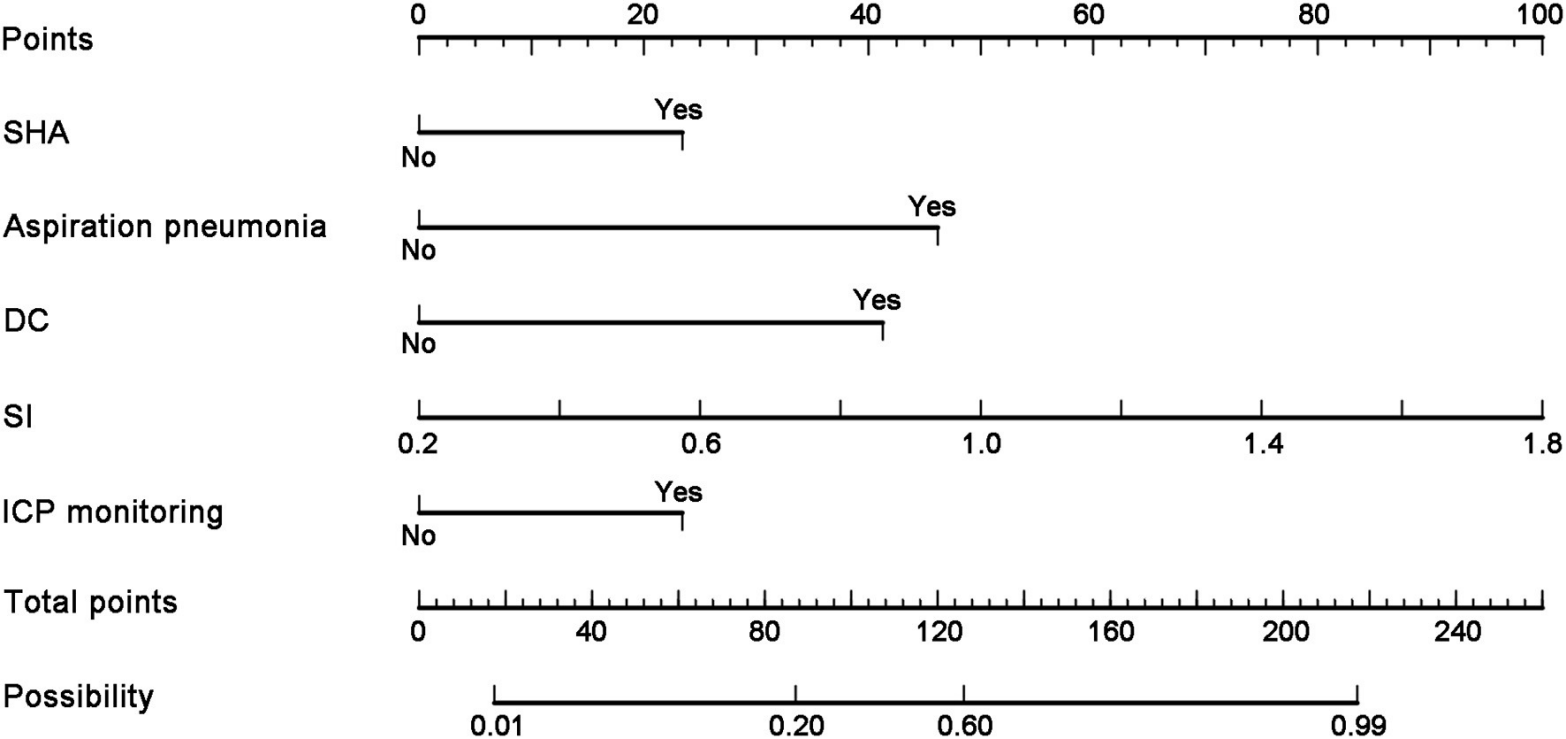
Corresponding score for the nomogram model. A nomogram model for predicting the risk of complicated cerebral infarction in patients with brain herniation was established by R software. The combined SAH score was 21.5 points, the combined aspiration pneumonia score was 46.5 points, and the decompression without craniectomy score was 41.5 points, and without ICP monitoring score was 21.5 points. For every 0.2 increase in the SI value, the nomographic score increased by 11.1 points.

The independent effects of these five items were monitored according to whether patients had SAH, aspiration pneumonia, DC, SI, and ICP monitoring. The factors were analyzed, and a nomogram model for predicting the risk of PTCI in patients with brain herniation was established by R software. According to the nomogram, the SAH score was 21.5 points, the aspiration pneumonia score was 46.5 points, and absence of DC had a score of 41.5 points. For every 0.2 increase in the SI value, the nomographic score increased by 11.1 points, and the absence of ICP monitoring had a score of 21.5 points. A high score in the line graph model was associated with a higher risk of PTCI after brain herniation (Figure 4, Table 5).

**Table 5:** A nomogram model score for predicting the risk of PTCI in patients with brain herniation.

**Variable in nomogram**	**Nomogram score**
SAH	21.5
Aspiration pneumonia	46.5
DC	41.5
SI	11.1/0.2
ICP monitoring	21.5

*SAH, subarachnoid hemorrhage; DC, decompressive craniectomy; SI, shock index; ICP, intracranial pressure*.

*The line score plot increased by 11.1 points for each 0.2 increase in SI values and by 21.5 points for no ICP monitoring*.

**Figure 5:**
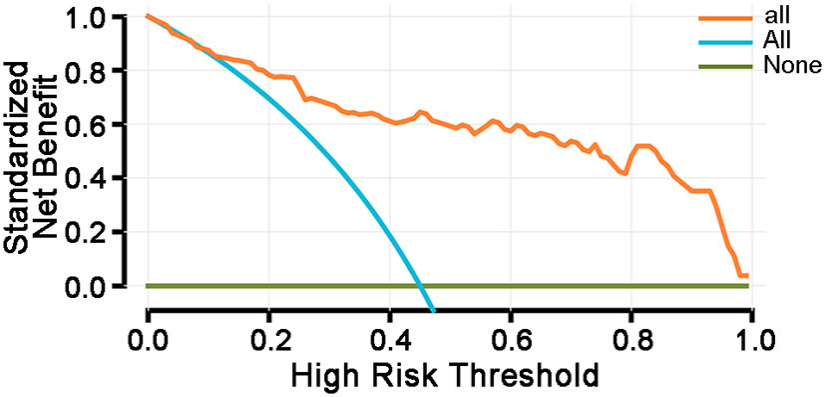
Decision curve analysis. Survival rates (net benefit rates) were compared for three different decisions. The three curves represent: (1) all patients have no factors that influence the risk of developing PTCI (represented by NONE, horizontal line), (2) all patients are at risk of developing PTCI (represented by ALL, slash), and (3) he decision curve of this study. This prediction model can be used for clinical decision-making. Thresholds are in the range of <85%, and the model has a higher survival rate (net benefit rate) relative to ALL or NONE.

Decision curve analysis was conducted to evaluate the predictive value of the nomogram model for PTCI in patients with cerebral herniation. At present, it is difficult to show PTCI in the early stage of imaging examination. The patients with craniocerebral trauma admitted to the Neurosurgery Department of the 900th Hospital between January 2018 and January 2020 were used as the verification population. According to the construction process of the decision curve, R software was used to calculate the net benefit under each threshold probability and a curve was generated. Before interpretation of the decision curve, it was assumed that the influencing factors had a predictive effect on the occurrence of PTCI. The study range of incidence was set in the interval 0–1.0 (Figure 5).

## Discussion

In this study, we retrospectively analyzed the patients with TBI and cerebral herniation who presented with PTCI admitted to our department between January 2018 and January 2020. Analysis of various correlated factors revealed that the GCS score at admission, SAH, ICP monitoring, DC, aspiration pneumonia, DC, SI, MAP, and GLU were significantly correlated with PTCI. Among them, SI was the primary influencing factor, which was consistent with the results of the classification tree model. Then, a nomogram model of the PTCI population was constructed. The total evaluation score was 240 points, and the observed score was 216 points, indicating a high predictive rate. The nomogram could intuitively display the possible influences screened by the logistic regression model and gave a quantitative risk assessment score. Timely intervention for PTCI-related risk factors early after TBI can help to reduce the probability of PTCI occurrence and thus improve the prognosis of TBI patients.

TBI patients are often associated with multiple injuries, and hypovolemic shock often already occurs before admission (9). SI is a commonly used assessment index for patients with acute trauma in clinical practice which can reflect the severity of trauma in patients (10). It was first described by Allgower and Burri in 1967, and has been reported to be a more sensitive marker of shock and of the likelihood of success of resuscitation efforts than traditional vital signs alone. SI is easily calculated at the bedside without the need for additional information or equipment, and it has also been used to identify mortality and the need for massive transfusion (11). When SI > 1.0, this indicates that the body's effective circulating blood volume is insufficient and the organs are in a state of ischemia and hypoxia. Persistent volume depletion easily leads to abnormal cerebral blood flow autoregulation, resulting in further limitation of cerebral blood perfusion (12). Aggravation of ischemia and hypoxia is a prerequisite for the development of cerebral infarction, and our statistical results confirmed that SI is a key risk factor for the development of PTCI. Therefore, the SI should be closely monitored during the treatment of patients with TBI.

Combined with the conclusions of this study, other possible causes of PTCI formation were further analyzed. In the early stage of brain herniation, the ICP increases under the influence of subhematoma, epidural hematoma compression, brain contusion and laceration, diffuse axonal injury, and arachnoid hemorrhage. The cerebral venous system is the first to be compressed, resulting in smaller lumens, slower blood flow, and progressive aggravation of edema. It may even cause temporary occlusion of the cerebral venous system or cerebral venous thrombosis (13). Cerebral congestion secondary to cerebral venous return disorder further aggravates cerebral ischemia and hypoxia (14). The sustained increase in ICP due to multiple factors leads to compression of the cerebral arterial system and reduced cerebral blood supply, resulting in ischemic infarction of brain tissue. In addition, cerebral tissue hypoxia can also lead to vasospasm and hence impaired cerebral blood flow (15). Moreover, cerebral infarction occurs when cerebral blood flow in the terminal vascular supply area is less than normal for more than 60–90 min.

DC is usually selected for surgery in TBI patients with brain herniation (16). All patients included in our study underwent surgery, including 63 patients who did not undergo DC. These patients underwent evacuation of intracranial hematoma or brain lobectomy. After operation, the brain tissue collapsed significantly and there was no cerebral edema. Therefore, we did not perform DC. However, the results showed that DC was an important prognostic factor in TBI patients with brain herniation, and the degree of intraoperative brain tissue swelling could not be used alone to determine whether DC surgery was performed. Studies have shown that decompressive craniectomy is not only effective in relieving malignant intracranial hypertension, but also in preventing and reversing brain herniation, as well as reducing the mortality of PTCI patients (17–19). However, during surgery using traditional methods, a sudden drop of ICP restores the blood flow of the arteriovenous system, and the local brain tissue volume increases dramatically together with diffuse brain swelling, leading to difficulty in closure of the cranium. Restriction of blood flow supply due to recompression of the cerebrovascular system by elevated ICP secondary to diffuse brain swelling is one of the causes of PTCI. Decompressive craniectomy after cerebral infarction can significantly reduce the risk of death (19).

Statistical analysis in this study showed that a lower GCS score was associated with more severe intracranial injury (20), as indicated by the brain computed tomography (CT) imaging results. Epidural and subdural hematoma, diffuse SAH, brain contusion, brain stem injury, and diffuse axonal injury were more likely to coexist as multiple injury types. The earlier and faster the formation of brain herniation, the higher the probability of PTCI. Currently, the generally recognized pathogenic mechanism is an obvious displacement of brain tissue after brain herniation, with compression and stretching of blood vessels, resulting in massive cerebral infarction. For example, herniation of the tentorial notch can easily lead to blood flow obstruction in the ipsilateral occipital lobe, which is mainly dominated by the posterior cerebral artery. Subfalcine herniation can easily lead to central paralobular superior frontal gyrus and cingulate gyrus cerebral infarction. Some studies have also shown that the incidence of vasospasm in patients with SAH after craniocerebral trauma was positively correlated with the degree of injury (21). Latronico and Marino (22) confirmed by cerebral angiography that the incidence was as high as 5–57%. KREITER (23) and coworkers showed that after traumatic SAH, the release of a large number of vasoactive substances (including catecholamines and their decomposition products thromboxane, hemoglobin, etc.) could stimulate the formation of blood clots in the blood vessel wall. The clots and possible changes in blood flow can activate thrombin C, which in turn activates protein kinase C, leading to aggravation of vasospasm and microcirculation disturbances, finally causing cerebral infarction. There was a significant correlation between SAH and the size of the clot and cerebral vasospasm, and significant cerebral ischemia and even cerebral infarction could occur when the vessel caliber is reduced by more than 60%. Therefore, secondary vasospasm in TBI patients with SAH was one of the important factors leading to PTCI, and treatment to prevent vasospasm may reduce the incidence of PTCI.

With the advancement of medicine, more and more advanced monitoring technologies have been applied to the treatment of patients with TBI. In recent years, ICP monitoring technology has significantly improved the treatment of TBI patients (3, 24, 25). By accurately and continuously measuring the ICP throughout the treatment process, calculating the cerebral perfusion pressure, and adjusting the MAP to control the cerebral perfusion pressure at 60–70 mmHg, blood perfusion and compliance of cerebral tissue can be ensured, and the aggravation of ischemia and hypoxia can be avoided (26). At the same time, this also guides clinical precision medicine (27), and plays an important guiding role in the treatment of PTCI and cerebral herniation. ICP monitoring here refers to ICP monitoring of the ventricle type (28). The pressure is measured in real-time, and the ICP is controlled within a reasonable range by releasing cerebrospinal fluid (29). This is also one of the means to reduce the incidence of cerebral infarction. Interventions that precede irreversible brain damage in patients with cerebral infarction were significantly different in the control group of the present study and had a deterministic effect on improving prognosis. ICP monitoring was not performed in some patients in our study. The main reason is that some patients did not accept the side effects of ICP monitoring, such as infection, bleeding, tube blockage, and puncture failure. In addition, patients refused to use ICP monitoring for financial reasons. Therefore, these patients only underwent evacuation of intracranial hematoma or DC. The incidence of PTCI was higher in patients who did not undergo ICP monitoring.

This study was a single-center study, and our results need to be confirmed by multicenter studies with larger sample sizes, which will be our next research focus. In addition, all data were obtained before surgery, and we did not analyze intraoperative and postoperative data. Further analysis of intraoperative and postoperative data may result in the identification of more accurate indicators for cerebral infarction.

## Conclusions

In conclusion, we analyzed the risk factors of PTCI to TBI complicated with brain herniation. The GCS score at admission, no decompressive craniectomy, no ICP monitoring, combined SAH, combined aspiration pneumonia, SI, MAP, and high GLU were risk factors for infarction, of which SI was the primary predictor of PTCI in TBI. Timely intervention based on PTCI-related risk factors can help to reduce the probability of PTCI occurrence and thus improve the prognosis of TBI patients. In addition, besides treating the cause of cerebral infarction in patients with cerebral infarction, reducing bleeding and actively improving circulation was also an important method to improve prognosis. Different from other cerebral infarction formation mechanisms, PTCI after cerebral herniation was a transient ischemic change. Better and faster intervention measures can be taken before irreversible brain damage occurs in patients with cerebral infarction. Accurate judgment of disease outcome will be the focus of future research.

## Data availability statement

The original contributions presented in the study are included in the article/supplementary material, further inquiries can be directed to the corresponding author/s.

## Ethics statement

The studies involving human participants were reviewed and approved by the Ethics Committee of the 900th Hospital. The patients/participants provided their written informed consent to participate in this study.

## Author contributions

XH: conceptualization, formal analysis, methodology, and writing—original draft. JT and JX: data curation. SZ: formal analysis and methodology. LZ: methodology. LW: formal analysis and writing. SW: project administration and writing—review and editing. All authors have read and agreed to the published version of the manuscript, contributed to the article, and approved the submitted version.

## Funding

The present study was funded by the Fujian Provincial Science and Technology Innovation Joint Fund (2019Y9045).

## Conflict of interest

The authors declare that the research was conducted in the absence of any commercial or financial relationships that could be construed as a potential conflict of interest.

## Publisher's note

All claims expressed in this article are solely those of the authors and do not necessarily represent those of their affiliated organizations, or those of the publisher, the editors and the reviewers. Any product that may be evaluated in this article, or claim that may be made by its manufacturer, is not guaranteed or endorsed by the publisher.
